# Recovered frog populations coexist with endemic *Batrachochytrium dendrobatidis* despite load‐dependent mortality

**DOI:** 10.1002/eap.2724

**Published:** 2022-10-27

**Authors:** Matthijs Hollanders, Laura F. Grogan, Catherine J. Nock, Hamish I. McCallum, David A. Newell

**Affiliations:** ^1^ Faculty of Science and Engineering Southern Cross University Lismore New South Wales Australia; ^2^ Centre for Planetary Health and Food Security, School of Environment and Science Griffith University Gold Coast Queensland Australia

**Keywords:** amphibian, *Batrachochytrium dendrobatidis*, chytridiomycosis, continuous‐time, disease, hidden Markov model, infection intensity, mark‐recapture

## Abstract

Novel infectious diseases, particularly those caused by fungal pathogens, pose considerable risks to global biodiversity. The amphibian chytrid fungus (*Batrachochytrium dendrobatidis*, *Bd*) has demonstrated the scale of the threat, having caused the greatest recorded loss of vertebrate biodiversity attributable to a pathogen. Despite catastrophic declines on several continents, many affected species have experienced population recoveries after epidemics. However, the potential ongoing threat of endemic *Bd* in these recovered or recovering populations is still poorly understood. We investigated the threat of endemic *Bd* to frog populations that recovered after initial precipitous declines, focusing on the endangered rainforest frog *Mixophyes fleayi*. We conducted extensive field surveys over 4 years at three independent sites in eastern Australia. First, we compared *Bd* infection prevalence and infection intensities within frog communities to reveal species‐specific infection patterns. Then, we analyzed mark‐recapture data of *M. fleayi* to estimate the impact of *Bd* infection intensity on apparent mortality rates and *Bd* infection dynamics. We found that *M. fleayi* had lower infection intensities than sympatric frogs across the three sites, and cleared infections at higher rates than they gained infections throughout the study period. By incorporating time‐varying individual infection intensities, we show that healthy *M. fleayi* populations persist despite increased apparent mortality associated with infrequent high *Bd* loads. Infection dynamics were influenced by environmental conditions, with *Bd* prevalence, infection intensity, and rates of gaining infection associated with lower temperatures and increased rainfall. However, mortality remained constant year‐round despite these fluctuations in *Bd* infections, suggesting major mortality events did not occur over the study period. Together, our results demonstrate that while *Bd* is still a potential threat to recovered populations of *M. fleayi*, high rates of clearing infections and generally low average infection loads likely minimize mortality caused by *Bd*. Our results are consistent with pathogen resistance contributing to the coexistence of *M. fleayi* with endemic *Bd*. We emphasize the importance of incorporating infection intensity into disease models rather than infection status alone. Similar population and infection dynamics likely exist within other recovered amphibian‐*Bd* systems around the globe, promising longer‐term persistence in the face of endemic chytridiomycosis.

## INTRODUCTION

The unprecedented global emergence of fungal pathogens is increasingly threatening human and ecosystem health (Fisher et al., 2020). Amphibian chytridiomycosis, caused by fungal agents *Batrachochytrium dendrobatidis* (*Bd*) in frogs and *B. salamandrivorans* (*Bsal*) in salamanders, has decimated amphibian communities around the world (Berger et al., 1998; Scheele et al., 2019). *Batrachochytrium dendrobatidis* has contributed to the decline of ~6.5% of frog species, including 90 presumed extinctions, which represents the greatest loss of vertebrate biodiversity attributable to a pathogen (Scheele et al., 2019). Not all affected species have gone extinct, however, and vastly different patterns of decline have been observed, from extinctions to slower ongoing declines (Scheele et al., 2019), stabilization at lower abundance (Briggs et al., 2010), and crash and recovery (Newell et al., 2013; Phillott et al., 2013; Retallick et al., 2004).

Many frog species that initially declined following the emergence of *Bd* have since experienced recoveries (Briggs et al., 2010; Knapp et al., 2016; Newell et al., 2013; Phillott et al., 2013; Retallick et al., 2004; Scheele et al., 2017). Although virulence of the pathogen may attenuate (Langhammer et al., 2013), coexistence of recovered frog populations with endemic *Bd* does not necessarily imply that the fungus has become innocuous (Brannelly et al., 2021; Scheele et al., 2017). Several field studies estimating post‐epidemic disease impacts using marked animals found that *Bd* negatively impacted frog survival even in stable populations (Grogan et al., 2016; Murray et al., 2009; Muths et al., 2011; Muths et al., 2020; Spitzen‐van der Sluijs et al., 2017). Other studies report limited ongoing impact of *Bd* on survival probabilities in frog populations after initial declines (Briggs et al., 2010; Retallick et al., 2004). Additionally, disease impact is influenced by a range of factors, including seasonal environmental factors which may relatively favor the pathogen over the host (Kriger & Hero, 2007; Phillott et al., 2013; Stevenson et al., 2013). Post‐epidemic pathogen impact is thus highly species and context dependent, and mechanisms that permit coexistence are often unclear (Brannelly et al., 2021).

Methodological differences may also lead to contrasting inferences about the impacts of endemic *Bd*. For example, most studies assessing disease susceptibility have focused on the effect of *Bd* infection status (infected or uninfected). Many of these studies implicitly apply the SIR paradigm in disease ecology (Anderson & May, 1992), assuming that the fundamental characteristics of a host‐pathogen system can be captured by considering hosts as susceptible, infected, or recovered. However, in many infectious disease systems, including fungal pathogens such as *Bd* (Langwig et al., 2016; Wilber et al., 2021), mortality risk appears to be dependent on infection intensity (pathogen load), and intensity is likely to be a more accurate predictor of potential survival costs associated with infection (Grogan et al., 2016; Heard et al., 2014; Joseph & Knapp, 2018; Spitzen‐van der Sluijs et al., 2017).

Incorporating infection intensity as a time‐varying individual covariate in mark‐recapture models is challenging with frequentist methods used in most previous studies (Grogan et al., 2016; Murray et al., 2009), but such models are readily fitted using Bayesian approaches incorporating Markov chain Monte Carlo (MCMC) methods. Although covariates cannot contain missing values, submodels can be specified to impute these missing values which the model will treat as parameters to be estimated (Gelman et al., 2013). Here, we illustrate the potential of these methods to provide new insights into the effect of infection intensity on host population dynamics.

Mechanisms underlying population recoveries are difficult to determine after the event but can be classified as host immunological, behavioral changes at the individual or population level, pathogen attenuation, environmental factors, and changes in community composition (Brannelly et al., 2021). Intrinsic host defenses against disease have been separated into resistance and tolerance, where resistance describes a host's ability to limit pathogen burden, and tolerance describes a host's ability to persist with a pathogen despite carrying given pathogen burdens (Brannelly et al., 2021; Råberg et al., 2009). An indication of which mechanism is active in given species can be gathered by comparing infection intensities between sympatric species, where more resistant species are expected to have lower infection intensities (Råberg et al., 2009).

In this study, we aimed to explore and quantify the role of endemic *Bd* on now‐recovered frog communities several decades after putative disease‐associated declines. We focus on an Australian narrow‐range stream‐associated species, *Mixophyes fleayi* (Fleay's barred frog). Populations of this species recovered following a period of range‐wide declines during the epidemic wave of chytridiomycosis on the east coast of Australia, which likely started in Brisbane, Queensland, around 1978 (Murray et al., 2010; Newell et al., 2013). We conducted surveys of post‐metamorphic frogs for 4 years at three independent field sites to investigate community‐level infection patterns, *M. fleayi* susceptibility to chytridiomycosis, and infection dynamics. First, we compared *Bd* infection prevalence and intensity within the frog communities at each site to reveal species‐level responses and environmental factors influencing infection patterns. Then, we used a Bayesian multistate mark‐recapture model to estimate the effect of *Bd* infection status and infection intensity on mortality of adult *M. fleayi*, the effects of seasonal covariates on infection dynamics, and factors relating to frog detectability. Our study represents a significant contribution to assessing the infection dynamics and role of endemic *Bd* in amphibian populations that have recovered post disease‐associated declines.

## METHODS

### Study species

**FIGURE 1:**
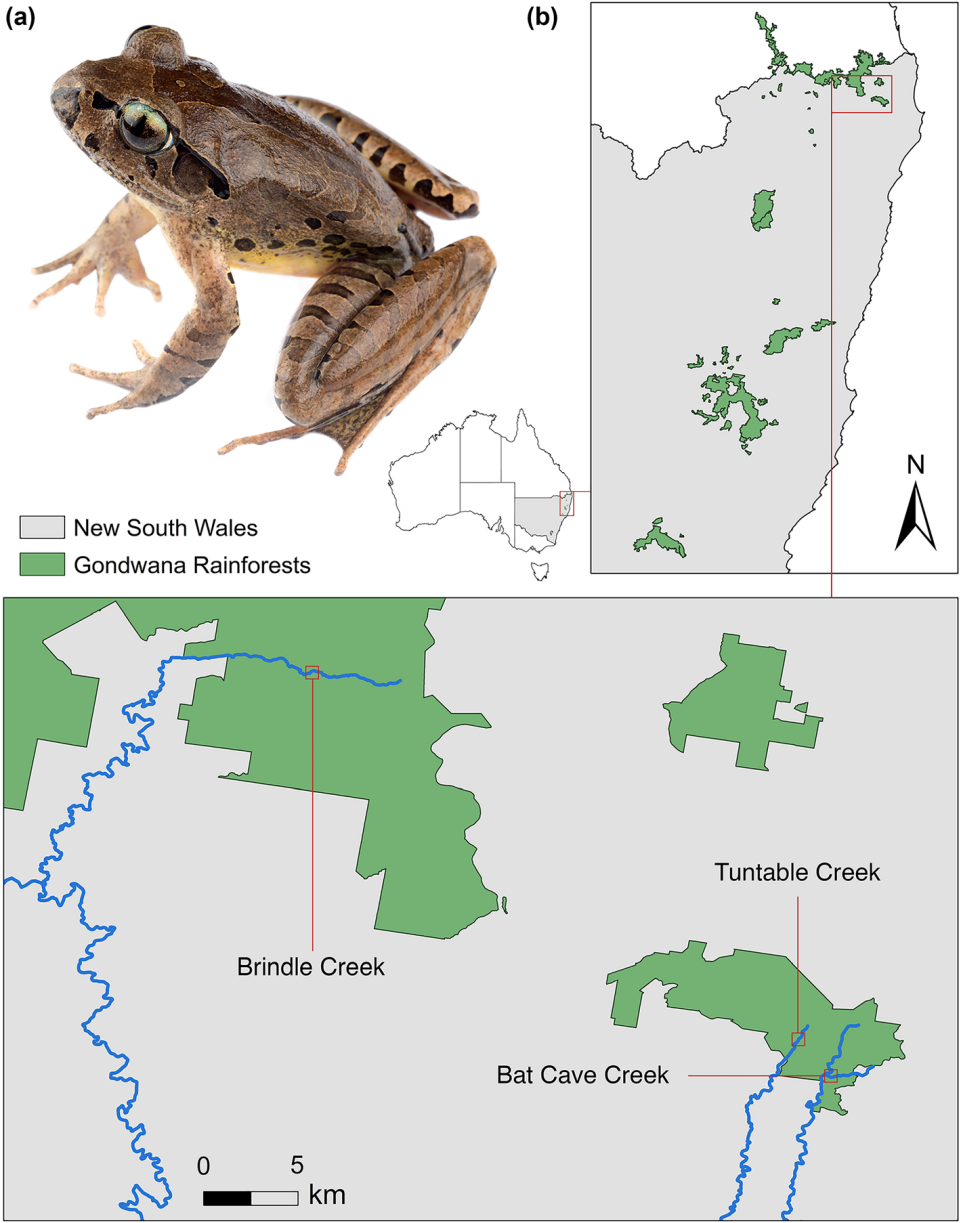
(a) Adult male of the study species *Mixophyes fleayi* (Fleay's barred frog). Note the subcutaneous passive integrated transponder tag near the urostyle. (b) Map of the study sites. Date source: Esri.

*Mixophyes fleayi* (Fleay's barred frog, Myobatrachidae) is endemic to the wet forests of southeast Queensland and northeast New South Wales, Australia (Figure 1a). It is a large (snout‐to‐urostyle length [SUL] 60–80 mm), obligate stream breeder (Stratford et al., 2010) and is currently listed as Endangered on the IUCN Red List. Males live by streams and display a high degree of site fidelity, while females are infrequently captured (Newell et al., 2013). Annual apparent survival probabilities in males range from 0.38–0.65 (Newell et al., 2013).

*Mixophyes fleayi* first suffered declines in the 1980s (Newell, 2018). Although declines occurred before the description of *Bd*, they coincided with the epidemic wave of *Bd*, and dead *M. fleayi* with chytridiomycosis were found in the 1990s (Berger et al., 1998; Newell et al., 2013). Surveys conducted later in this decade failed to detect the species at many historic localities and detected low numbers where they did persist (Goldingay et al., 1999; Newell, 2018). Recovery of some populations apparently started shortly thereafter, and an 8‐year mark‐recapture study initiated in 2000 at two sites in northern New South Wales revealed a 3‐ to 10‐fold increase in population size over the study period (Newell et al., 2013). Surveys conducted 5 years later indicated that abundance and survival remained stable (Quick et al., 2015). Nevertheless, *M. fleayi* appears to have been extirpated and not recovered from at least three locations (Newell, 2018).

Additional species incorporated in the study were *Litoria wilcoxii*, *L. pearsoniana*, and *Mixophyes iteratus*, of which the latter two were reported to experience *Bd*‐associated declines before apparent population recoveries.

### Study sites

Field surveys were conducted along stream transects at three independent rainforest sites (Brindle, Tuntable, and Bat Cave Creeks) in the Gondwana Rainforests of Australia World Heritage Area in northern New South Wales, Australia (Figure 1b). The region is subtropical and characterized by warm, wet summers and mild, dry winters. The sites were chosen over an elevation gradient because the effects of *Bd* in Australia vary considerably with elevation (Scheele et al., 2017), with Brindle, Tuntable, and Bat Cave creeks situated at 750, 450 m, and 200 m asl, respectively. Brindle and Tuntable Creeks were previously sampled for *M. fleayi* from 1997 to 2008 (Goldingay et al., 1999; Newell et al., 2013), with Brindle Creek subject to further sampling from 2013 to 2014 (Quick et al., 2015).

### Field surveys

We conducted 134 nocturnal mark‐recapture surveys over 4 years targeting *M. fleayi* from the austral spring of 2017 to the austral autumn of 2021. We commenced surveys on dark with two to three surveyors using headtorches and call playback to locate frogs at the stream and up to ~20 m from the water's edge. Frogs were captured using clean plastic bags, weighed, measured, and swabbed using sterile rayon‐tipped swabs (Medical Wire & Equipment Dryswabs MW100) to sample for *Bd*. Frogs with SUL > 40 mm were individually marked using subcutaneous passive integrated transponder (PIT) tags (Trovan ID‐100B/1.4) (Figure 1a), with entry points sealed with tissue adhesive (3M Vetbond) (Newell et al., 2013).

Surveys were conducted following the robust design (Pollock, 1982), with two to three secondary surveys (conducted on consecutive nights) per primary survey occasion, with 19–21 primary occasions per site. Individuals were swabbed once per primary occasion. From 2017 to 2019, three to five primary surveys per year were conducted per site from spring to autumn. From 2019 to 2021, primary surveys were conducted at 6‐week intervals throughout the year.

We additionally swabbed up to 15 individuals of each sympatric frog species encountered at each site for every primary survey occasion from 2019 to 2021. We collected these samples to estimate intercommunity variation of *Bd* infection prevalence and intensity and to investigate seasonal drivers of *Bd* infection. Site‐specific meteorological data were extracted from SILO, a database providing interpolated data on spatial grids (Jeffrey et al., 2001).

### *Bd* detection

DNA was extracted from swab tips with Prepman Ultra (Applied Biosystems) using a standard protocol (Brannelly et al., 2020; Hyatt et al., 2007) and quantified using qPCR—for details, see Appendix S1. We used gBlocks Gene Fragments (Integrated DNA Technologies) synthetic standards of the amplified partial ITS region of *Bd* to generate standard curves in each qPCR run. Samples were run in duplicate and considered positive when at least one well amplified >0 ITS copies (Briggs et al., 2010; Hyatt et al., 2007). We report *Bd* infection intensity as log_10_ ITS copies per swab because the ITS copy number of local *Bd* strains is unknown (Longo et al., 2013).

### Statistical analysis

#### Model implementation and reporting

We used Bayesian inference with NIMBLE 0.12.2 (de Valpine et al., 2017) through R 4.1.0 (R Core Team, 2021) to sample posterior distributions of model parameters using Markov chain Monte Carlo (MCMC) methods. We specified vague Beta(1, 1) and Exponential(1) priors on all transformed intercepts of probabilities and hazard rates, respectively, and weakly informative (half‐)Cauchy priors on all coefficients, standard deviations of random effects, and the intercept of *Bd* infection intensity (Gelman et al., 2008). We used Reversible Jump MCMC (RJMCMC) for predictor variable selection (Green, 1995). All covariates were centered and divided by two standard deviations to allow direct comparison of continuous and binary predictors. We ran four chains for each model from overdispersed initial values for 25,000 iterations after discarding the first 5000 as burn‐in, saving every 10th draw yielding 10,000 posterior draws per model. Convergence of parameters of both models was achieved based on potential scale reduction factors ($\hat{R}$) < 1.05 and visual inspection of traceplots. For details, see Appendix S1.

We followed recently proposed guidelines for reporting posterior distributions (Makowski et al., 2019). Estimates are reported as medians and 95% highest density intervals (HDI). Effects were considered present when the probability of direction (*pd*, analogous to frequentist *p*‐values) was >97%. Effects were considered significant (i.e., whether an effect was strong enough to be considered meaningful) when <2.5% of the full posterior distribution was in the Region of Practical Equivalence (ROPE), defined as the interval [−0.5, 0.5] for (log)normal models and [−0.9, 0.9] for logistic models with covariates standardized to two standard deviations (Makowski et al., 2019). We additionally report RJMCMC inclusion probabilities, the percentage of MCMC iterations for which predictors were included in the model by RJMCMC. Differences between intercepts (species and sites) were considered significant when 95% HDIs of the differences did not overlap 0. Intercepts are reported on the original scale (e.g., probability, hazard rate) for ease of interpretation, and coefficients and random effects are reported on the link function scale (e.g., logit, log). Figures show “model‐averaged” effects of predictors, including where RJMCMC excluded the effect and toggled coefficients to 0.

#### *Bd* infection prevalence and intensity in the frog communities

To estimate species‐level *Bd* prevalence and infection intensity, we fitted logistic and lognormal regression models to infection status and infection intensity data, respectively, on species with at least 10 positive samples. We modeled log_10_ infection intensity with a lognormal distribution due to the remaining overdispersion after log_10_‐transforming the values. Expected prevalence and intensity were modeled as functions of species‐level intercepts, average daily temperature, and average 6‐weekly rainfall over the primary survey occasion intervals, and random site, survey, and individual effects. We calculated Bayesian *p*‐values from Pearson's χ^2^ statistics for each model.

#### Mark‐recapture analysis

We fit a robust design multistate Arnason‐Schwarz model to the *M. fleayi* mark‐recapture data to estimate individual mortality rates dependent on *Bd* infection status and intensity, *Bd* infection state transition rates, and individual recapture probabilities (Kéry & Schaub, 2012; Schwarz et al., 1993). We specified a continuous‐time formulation of the ecological process, parameterized with a transition rate matrix of hazard rates (hereafter, rates) for infection state transitions and mortality, to account for unequal primary occasion intervals and to model these processes more appropriately (Conn et al., 2012; Ergon et al., 2018; Glennie et al., 2022; Miller & Andersen, 2008). The robust design facilitated improved inference by modeling the detection process at the level of secondary surveys. Mortality is “apparent mortality” because mortality is confounded with permanent emigration from the study area. To accommodate differences in number of surveys between sites, capture histories of some sites were padded with empty columns to be imputed from the joint posterior distribution. Full code is available in Hollanders et al. (2022b).

We modeled apparent mortality rates as log‐linear functions (Ergon et al., 2018) of body condition (scaled mass index, Peig & Green, 2009), average daily temperature over primary occasions (a proxy for season), *Bd* infection status, *Bd* infection intensity, and all pairwise interactions as predictors. Infection state transition rates were modeled as log‐linear functions of body condition, average daily temperature and average 6‐weekly rainfall over the primary interval, their interaction, and *Bd* infection intensity, under the assumption that individuals did not change infection state more than once during a 6‐week interval. Recapture probabilities were modeled as logit‐linear functions of body condition, sex, daily temperature on the survey day, average 6‐weekly rainfall over the primary interval, their interaction, *Bd* infection status, and *Bd* infection intensity. We additionally included random individual effects to account for heterogeneity in recapture probabilities due to differences in individual home ranges, transience, and position on the transect. Results and discussion of the recapture probabilities are reported in Appendix S2. Mortality rates, infection state transition rates, and recapture probabilities were modeled with site‐specific intercepts and random temporal (survey) variation. Missing body condition and infection intensity values for all primary occasions that individuals were not captured were imputed with random individual effects using MCMC. Imputation of infection intensity additionally included seasonal effects of temperature and rainfall.

We assessed model fit with two discrepancy statistics, inspired by Rankin et al. (2016). For the first, we counted observed infection state transitions between consecutive primaries for each individual to gauge the fit of our state process (Kéry & Royle, 2020). For the second, we tallied occasions each individual was recaptured as either uninfected or infected with *Bd* to infer fit of the detection model. For details, see Appendix S1.

## RESULTS

### Sampling summary

**FIGURE 2:**
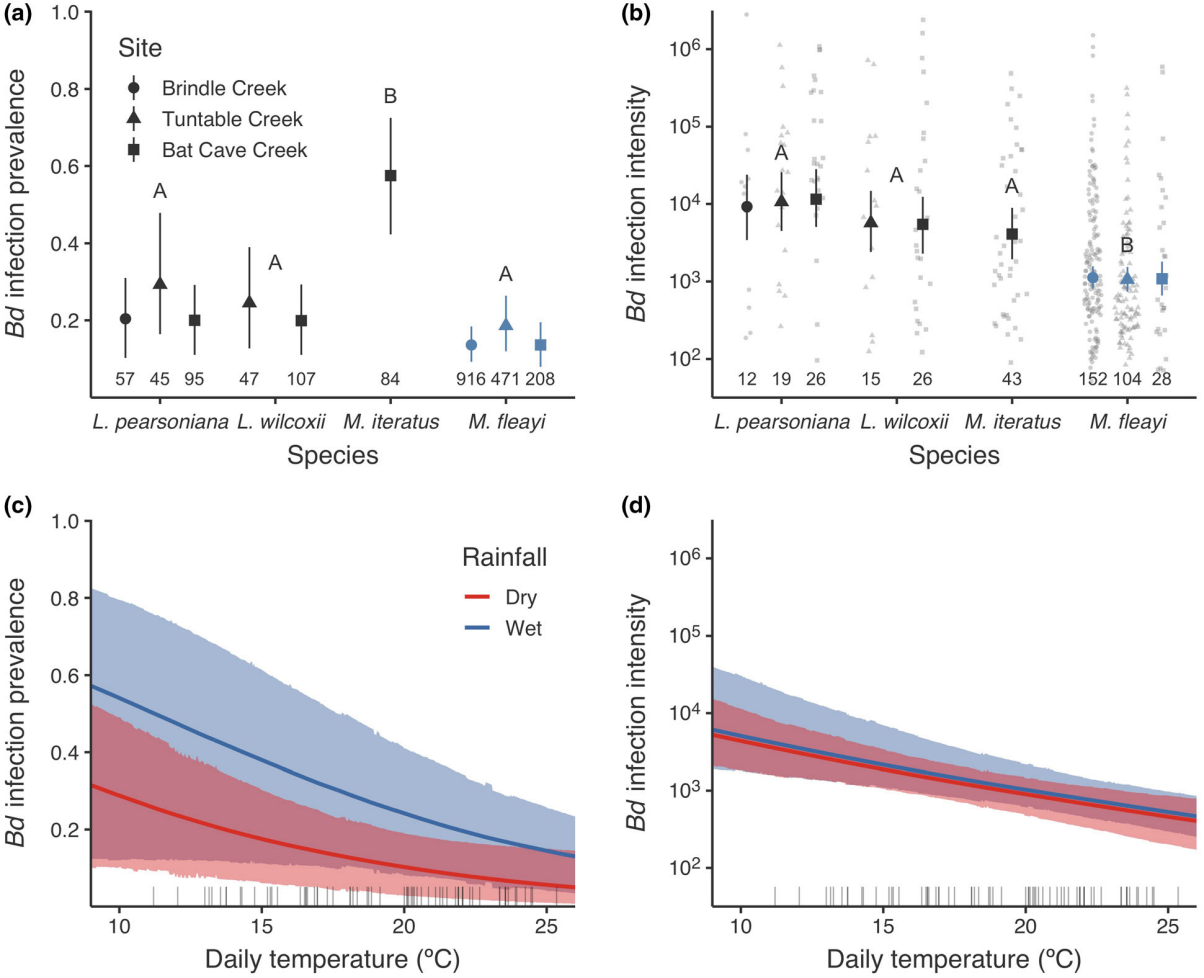
Species‐by‐site estimates (median and 95% highest density intervals [HDI]) of (a) *Bd* infection prevalence and (b) *Bd* infection intensity (ITS copies per swab). Transparent points in (b) are observed infection intensities. Letters indicate species differences, considered significant when 95% HDIs of intercepts did not overlap 0. Numbers along *x*‐axes in (a) and (b) are site‐level sample sizes for each model. Blue was used to highlight *Mixophyes fleayi*. Prediction curves (median and 95% HDI) are the effects of average interval temperature and *average 6‐weekly* interval rainfall on (c) *Bd* infection prevalence and (d) *Bd* infection intensity, using *M. fleayi* as intercepts. Dry and wet rainfall periods correspond to no rainfall and 575 mm (approximately two standard deviations above the observed mean of 195 mm) over 6‐week survey intervals. Rug plots show observed average daily temperatures of primary survey occasion intervals.

We captured 686 unique *Mixophyes fleayi* and collected 487 swab samples from eight other species across the three sites. Of those, three species (*Litoria pearsoniana*, *L. wilcoxii*, and *Mixophyes iteratus*) returned at least 10 positive *Bd* samples (see Figure 2a for sample sizes). Of all *M. fleayi* captured, 462 were at Brindle Creek, 136 at Tuntable Creek, and 88 at Bat Cave Creek. The majority (86%) were males. At each site, some individuals captured during the first occasion were also captured during the last occasion, indicating some individuals were known to be alive throughout the 4‐year study. We made 1609 primary detections (2384 including recaptures during secondary surveys), of which 248 (15%) involved a frog infected with *Bd*. Loads were overdispersed and ranged from 1.425 to 6.18 log_10_ ITS copies per swab, with the latter recorded on a female in winter with a diminished righting reflex, indicating poor health. We made 118 observations of frogs gaining *Bd* infection and 124 observations of frogs clearing infection. Individuals were observed to gain infections up to three times and to clear infections up to four times during the study period. For a graphical summary of observed *M. fleayi* captures, prevalence, and infection intensity per primary occasion, see Appendix S3: Figure S1.

### *Bd* infection prevalence and intensity in the frog communities

There were significant differences in both *Bd* infection prevalence and intensity within frog communities at the three sites (Figure 2a,b, Appendix S3: Table S1). *Mixophyes iteratus* had significantly higher prevalence (0.575 [0.42, 0.722]) than *L. pearsoniana* (0.229 [0.121, 0.395]), *L. wilcoxii* (0.223 [0.107, 0.417]), and *M. fleayi* (0.152 [0.065, 0.272]) (Figure 2a). Daily temperature and cumulative rainfall over 6 weeks prior to sample collection significantly predicted infection prevalence, with a negative effect of temperature (log odds change −1.025 [−1.631, −0.449], *pd* = 100%, 0.1% in ROPE, 93.4% inclusion) and a positive effect of rainfall (log odds change 0.75 [0.212, 1.314], *pd* = 99.5%, 1% in ROPE, 86.7% inclusion) (Figure 2c). The Bayesian *p*‐value for the prevalence model was 0.535.

Infection intensity of *M. fleayi* (3.036 [2.836, 3.234]) was significantly lower than that of *L. pearsoniana* (4.014 [3.636, 4.402]), *L. wilcoxii* (3.748 [3.337, 4.175]), and *M. iteratus* (3.614 [3.287, 3.949]) (Figure 2b). There was a negative and significant effect of temperature on infection intensity (log change −0.163 [−0.247, −0.089], *pd* = 100%, 0.1% in ROPE, 98.9% inclusion) (Figure 2d). The effect of rainfall was weakly positive when included in the model (log change 0.078 [0.013, 0.143], *pd* = 98.5%, 20% in ROPE, 26.2% inclusion). The Bayesian *p*‐value for the intensity model was 0.44.

### Mark‐recapture analysis

#### Apparent mortality rates

**FIGURE 3:**
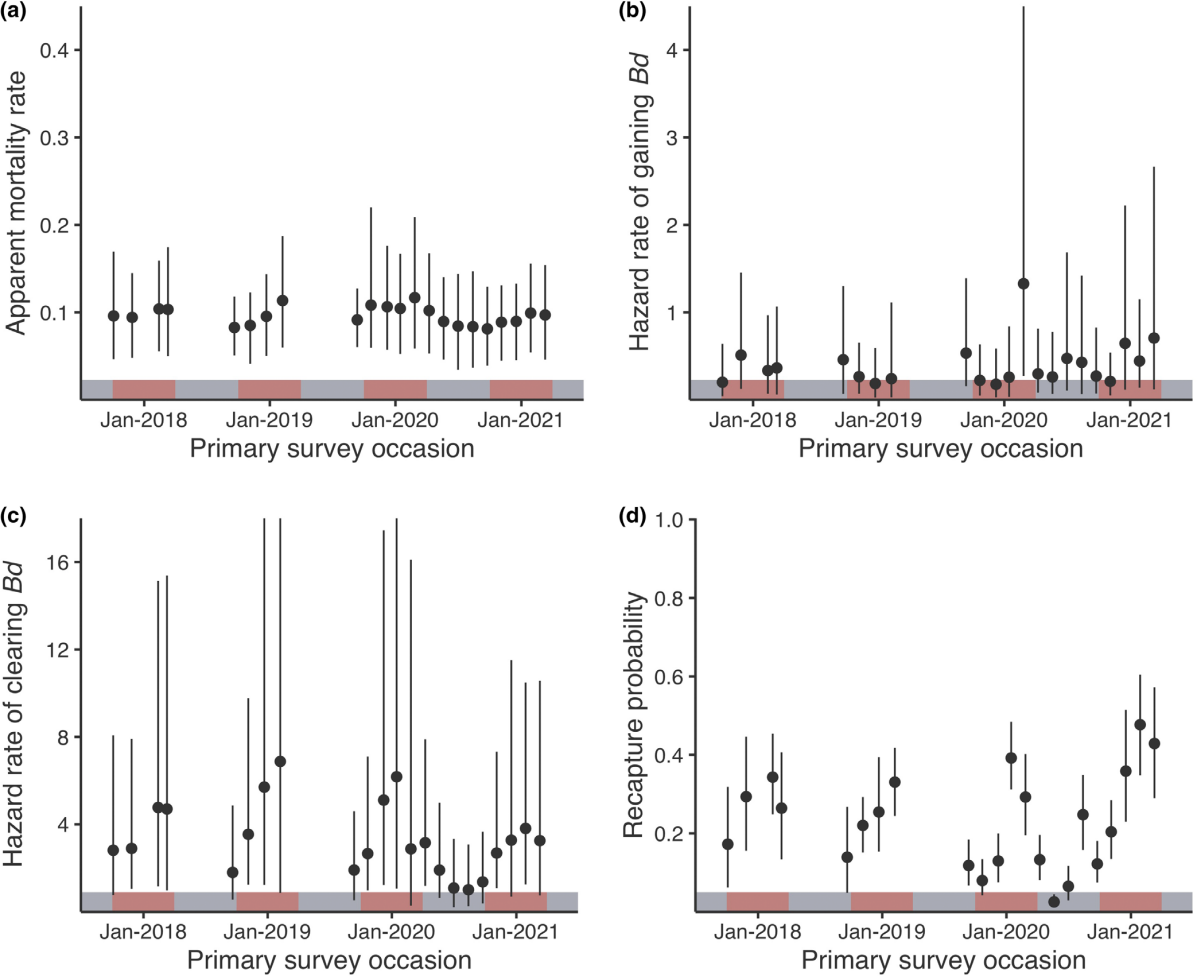
Site‐averaged estimates (median and 95% highest density intervals [HDI]) of *Mixophyes fleayi* (a) 6‐weekly apparent mortality rate, (b) 6‐weekly hazard rate of gaining *Bd* infection, (c) 6‐weekly hazard rate of clearing *Bd* infection, and (d) recapture probability per primary survey occasion. Red and gray demarcations indicate austral summer (October–April) and winter (May–September), respectively.

**FIGURE 4:**
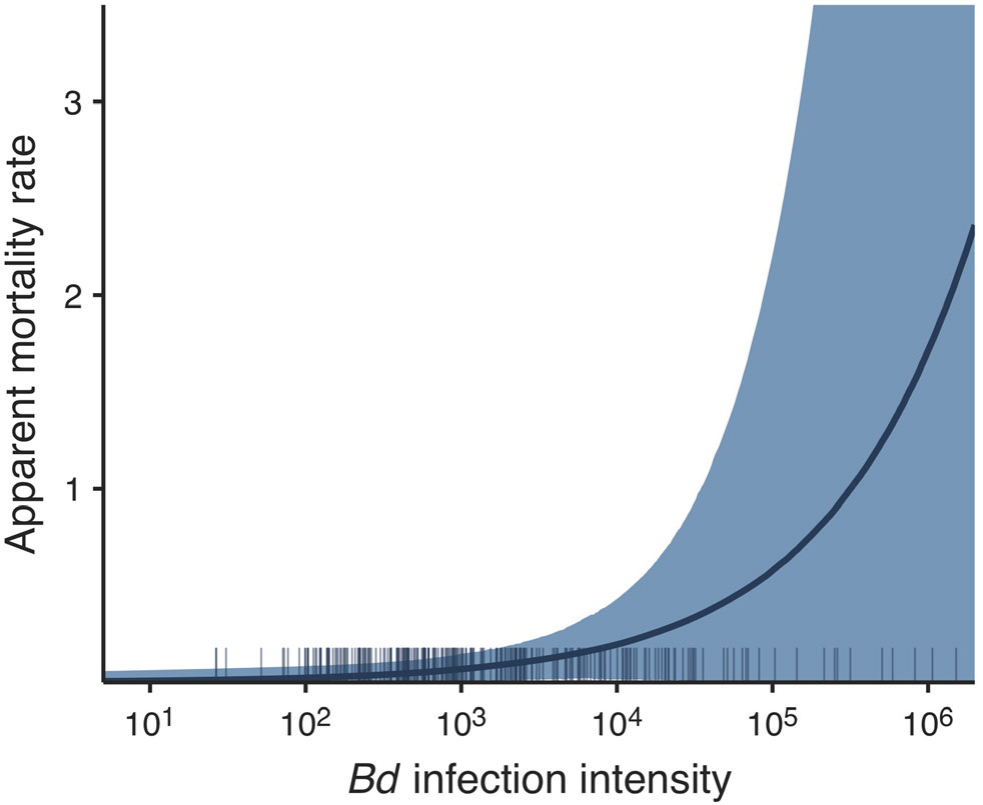
Prediction curve (median and 95% highest density intervals [HDI]) of the effect of *Bd* infection intensity (ITS copies per swab) on *Mixophyes fleayi* 6‐weekly apparent mortality rate. The rug plot shows observed *Bd* infection intensities.

Median 6‐weekly mortality hazard rates for *M. fleayi* ranged from 0.077 to 0.104 (corresponding to 0.9–0.93 apparent survival probabilities; Appendix S3: Table S2), equating to an annual hazard rate of 0.783 [0.571, 0.997] (survival probability of 0.457 [0.364, 0.56]) averaged over the three sites. Note that mortality hazard rates can be interpreted as the expected number of deadly events over the 6‐week intervals. Mortality rates showed little temporal variation (standard deviation [SD] of random temporal effect on log hazard = 0.334 [0, 0.7]) and were highly constant year‐round (Figure 3a, Appendix S3: Figure S2a). There were no significant differences between sites. There was a weakly positive effect of body condition on mortality (log hazards change 0.414 [0.002, 0.823], *pd* = 97.2%, 4.7% in ROPE, 97.8% inclusion) and no effect of temperature (log hazards change 0.176 [−0.393, 0.686], *pd* = 73.3%, 97.3% inclusion) on apparent mortality. *Batrachochytrium dendrobatidis* infection status did not have an effect on mortality (log hazards change −0.167 [−1.714, 1.053], *pd* = 60%, 79.4% inclusion), but *Bd* infection intensity was associated with increased mortality (log hazards change 1.953 [−0.011, 3.834], *pd* = 97.5%, 2.8% in ROPE, 99.9% inclusion) (Figure 4). There was no evidence for any interaction effects.

#### Infection dynamics

**FIGURE 5:**
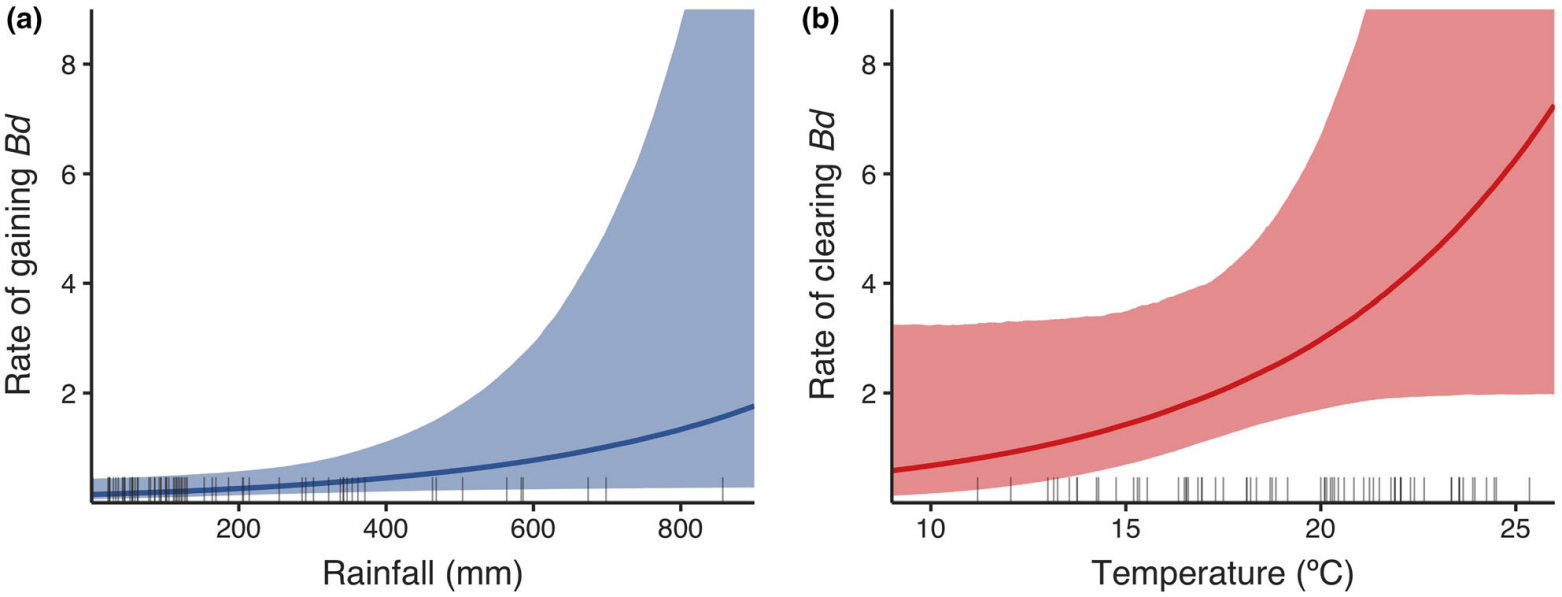
Prediction curves (median and 95% highest density intervals [HDI]) of the effects of (a) average 6‐weekly rainfall over the primary occasion interval on the hazard rate of *Mixophyes fleayi* gaining *Bd* infection and (b) average daily temperature over the 6‐weekly primary occasion interval on the hazard rate of *M. fleayi* clearing *Bd* infection. The intercepts of the curves are the means of the site‐specific intercepts. The rug plots show observed 6‐weekly average rainfall and average daily temperatures, respectively, of primary survey occasion intervals.

*Mixophyes fleayi* were less likely to gain *Bd* infections than to clear them over 6‐weekly intervals (Figure 3b,c, Appendix S3: Table S2). Median hazard rates of gaining infections ranged from 0.163 to 0.564 between sites (average 0.283 [0.12, 0.564], corresponding to a probability of 0.246 [0.129, 0.445]) and rates of clearing infections ranged from 2.418 to 2.892 (average 2.623 [1.308, 4.961], corresponding to a probability of 0.927 [0.817, 1]) over 6‐weekly intervals. There were no significant site differences in infection dynamics. There was no effect of body condition on the rates of gaining infection (log hazards change 0.138 [−0.703, 0.912], *pd* = 63.4%, 75.1% inclusion) and clearing infection (log hazards change −0.392 [−1.201, 0.351], *pd* = 85.3%, 81.1% inclusion). Infection dynamics showed seasonal patterns, with frogs more likely to gain infection in winter and more likely to clear infection in summer (Figure 3b,c, Appendix S3: Figure S2b,c). Rainfall was significantly positively associated with the rate of gaining infection (log hazards change 1.141 [−0.013, 2.253], *pd* = 98.1%, 2.5% in ROPE, 98.7% inclusion; Figure 5a), but we found no effect of average interval temperature (log hazards change −0.419 [−1.61, 0.79], *pd* = 76.4%, 93.2% inclusion). Conversely, there was a weakly positive effect of temperature on the rate of clearing infection (log hazards change 1.101 [−0.094, 2.302], *pd* = 96.4%, 4.2% in ROPE, 98.5% inclusion) (Figure 5b) but no effect of rainfall (log hazards change −0.246 [−1.42, 0.797], *pd* = 67.8%, 92.4% inclusion). Infection intensity had no effect on the rate of clearing infection (log hazards change 0.297 [−0.407, 1.024], *pd* = 80.3%, 75.6% inclusion). There was more unexplained temporal variation in rates of gaining infection (SD of random temporal effect on log hazard 0.492 [0.004, 0.981]) than rates of clearing infection (SD 0.29 [0.003, 0.786]).

#### Model fit

The Bayesian *p*‐value for the state process was 0.282, and for the detection model was 0.182, indicating good fit.

## DISCUSSION

In this study, we explored endemic chytridiomycosis infection dynamics and impacts on three rainforest stream‐associated frog communities over 4 years, with a particular focus on the recovered *Mixophyes fleayi* (Fleay's barred frog). We show that the amphibian chytrid fungus *Bd* does not appear to heavily impact *M. fleayi* populations 30 years after populations declined. Infection status alone did not predict mortality rates, but ongoing survival costs likely manifest through infrequent heavy infections, highlighting the importance of incorporating pathogen burdens in disease studies. Low *Bd* prevalence in *M. fleayi* is reflected in the low rates of gaining infection (average hazard rate of 0.283 on 6‐week intervals), and the relative rarity of high infection intensities likely results from *Bd* infections getting cleared at high rates (average rate of 0.927 on 6‐week intervals). Despite having declined sharply across its range during initial epidemics, the now‐recovered *M. fleayi* demonstrated the lowest *Bd* infection prevalence and intensities among sympatric frog species at our study sites. Our results are consistent with persisting *M. fleayi* populations demonstrating limited susceptibility and resistance to chytridiomycosis (Brannelly et al., 2021).

### Impact of *Bd* on individual mortality

By estimating apparent mortality hazard rates against observed infection intensities we demonstrated that *M. fleayi* with high infection burdens suffered increased mortality even though dead frogs were not observed in the field. For example, frogs carrying intensities of 10^5^ ITS copies per swab had an estimated median 4.4 times higher mortality rate than uninfected individuals, and frogs carrying 10^6^ ITS copies per swab experienced 11‐fold higher rates compared to uninfected individuals. The frog with the highest observed load, 10^6.18^ ITS copies per swab, showed a diminished righting reflex consistent with chytridiomycosis (Berger et al., 2005). However, infection intensities were overdispersed with high loads rarely observed (sensu Grogan et al., 2016). *Mixophyes fleayi* carrying the average observed *Bd* infection loads, 10^3.17^ ITS copies per swab, were not found to have increased apparent mortality. The constantly low estimates of mortality throughout the years suggest that unobserved mortality events (e.g., when frogs rapidly gain lethal infections and die between our surveys) did not occur. These results are consistent with endemic *Bd* having limited population‐level impact on recovered populations with a long history of *Bd* exposure (Knapp et al., 2016).

It is important to note that our sampled population consisted of 86% males and no juveniles, so that our results pertained to the adult male population at our study sites. Juveniles in particular are likely to have increased susceptibility to chytridiomycosis (Sauer et al., 2020), and further studies on differential infection impacts between life stages is warranted. Furthermore, females, which were underrepresented in the study, spend most time away from the streams where males live, in the surrounding ridges and forest. Although the habitat near the stream and on the surrounding ridges is similar, the sexes may be subjected to differing exposure rates depending on the distance to the streams. Due to the limited female sample size, we were unable to assess sex‐specific differences in *Bd*‐associated mortality.

The effect of *Bd* on *M. fleayi* mortality contrasts with the effect on a sympatric species, *Litoria pearsoniana*, where infection status alone was found to significantly decrease survival probabilities (Murray et al., 2009). Other studies have similarly failed to detect an effect of *Bd* infection status alone (Briggs et al., 2010; Phillott et al., 2013), or found responses to vary between species groups (Russell et al., 2019). Grogan et al. (2016) used mark‐recapture models with two infected states (low and high) and found equivocal results, where frogs with higher loads had lower survival than uninfected animals, but frogs with lower loads had higher survival than uninfected frogs.

The only field studies, to our knowledge, to include individual *Bd* infection intensity as a predictor on survival found that intensity was correlated with decreased survival, as in our current study (Heard et al., 2014; Joseph & Knapp, 2018; Spitzen‐van der Sluijs et al., 2017). Spitzen‐van der Sluijs et al. (2017) additionally tested for an effect of infection status on survival and found site‐specific effects, highlighting the importance of including both infection state and intensity in analyses. Our data suggests that *M. fleayi* is capable of surviving *Bd* infections until infection loads become too high, but that this is an uncommon event. We emphasize that the inclusion of infection intensity as a time‐varying individual predictor on apparent mortality provided a more nuanced insight into the cost of endemic *Bd* on frog populations, and we caution against making inferences based on infection status alone.

### Infection dynamics

We found significant differences in both *Bd* infection prevalence and intensity within frog communities at our three sites. Our focal species, *M. fleayi*, demonstrated low infection prevalence and infection intensities relative to sympatric species, suggesting it may be more resistant to *Bd* (Råberg et al., 2009). Interestingly, *M. iteratus*, a sympatric congeneric (endangered) species with similar behavior and using the same habitat as *M. fleayi* that is also undergoing population recoveries following putative *Bd*‐associated declines (Newell, 2018), had eight‐fold higher odds of being infected and 20% higher log_10_ infection intensity compared to *M. fleayi* at the one site where they co‐occur. While differing prevalence and intensities may be related to different levels of exposure to zoospores because of behavioral or habitat use differences, this is unlikely to be the case here, and host immunological defenses like tolerance or resistance may explain these differences (Brannelly et al., 2021). Identifying the mechanisms that may underlie these observed patterns in prevalence and infection intensities between species warrants further research (Brannelly et al., 2021). Differences in species‐level responses have been established in laboratory studies of amphibians, but targeted field studies to establish such data are lacking (Gervasi et al., 2013). Our modeling approach could expand to incorporate multiple species to formally test for differences in *Bd* impact, infection dynamics, and seasonal effects.

*Mixophyes fleayi* was observed to frequently gain and lose *Bd* infections, but the rate of clearing infections was always higher than the rate of gaining infections, consistent with some level of resistance to *Bd*. Infection dynamics were influenced by seasonal effects of temperature and rainfall, where infection prevalence and intensity were expected to peak in the austral winter (Kriger & Hero, 2007; Murray et al., 2009; Phillott et al., 2013; Stevenson et al., 2013). Infection prevalence and intensity were significantly higher in the frog communities during periods with lower temperatures and increased rainfall, with a stronger effect of temperature. Anecdotally, though no dead frogs were found during the study, the frog with the highest observed load was detected in August (austral winter). The rate of *M. fleayi* gaining infection increased with rainfall. For the rate of clearing infection, though the 95% HDIs of the coefficients overlapped 0 and were thus inconclusive (possibly due to the limited sample size of observed infection clearance), the effect of temperature trended negative, in line with the expectation. Our overall results were expected in the context of *Bd*'s preference for cooler and wetter conditions (Stevenson et al., 2013). Nevertheless, apparent mortality was not correlated with temperature, suggesting that increased prevalence and infection intensities in colder months had limited impact on mortality.

There appeared to be a difference in rates of gaining *Bd* infections between Tuntable and Bat Cave Creeks, suggesting that some effects relating to infection dynamics remained unmodeled. Although the 95% HDI of the difference overlapped 0, overall differences were apparent from plotted estimates (Appendix S3: Figure S2b). These two sites are in adjacent valleys and are likely to be genetically similar. We speculate that higher densities at Tuntable compared to Bat Cave Creek (with breeding congregations frequently observed at the former but never at the latter) may facilitate density‐dependent transmission (Briggs et al., 2010). Additionally, as Tuntable Creek is ~250 m higher than Bat Cave Creek, elevation may play a role as higher elevations are frequently associated with increased *Bd* prevalence (Scheele et al., 2017). However, higher rates of infection would consequently be expected at Brindle Creek, where the elevation is highest and the population density greatest, which we did not observe. As the conditions at Brindle Creek should be conducive to higher infection rates, we suggest that the lack of population connectivity between the Border Ranges (Brindle Creek) and Nightcap Ranges (Tuntable and Bat Cave Creeks) may have led to variation in the evolutionary response of these populations to chytridiomycosis, and hence, differing relationships between population density and infection rates. The genetic foundation of population‐level responses, in addition to potential genetic differences in the pathogen, is of great interest to further understand species responses to emerging diseases, and disentangling genetic and environmental factors may have great implications for management of threatened species (Savage et al., 2015). Assessing the frequencies of genetic markers associated with immunity between sites would further inform mechanisms of pathogen coexistence at these sites (Bataille et al., 2015; Brannelly et al., 2021).

## CONCLUSIONS

Overall, we found that *M. fleayi* populations that recovered post *Bd*‐associated declines were stable despite individuals accruing mortality risk with high infection intensities. The generally low infection prevalence and intensities, the lack of observed mortality events, limited impact of average infection intensity, and high rates of clearing infections suggest that *M. fleayi* is currently resistant to the amphibian chytrid fungus *Bd*. Nevertheless, the highest infection intensities were still associated with increased apparent mortality rates, highlighting the importance of inferring pathogen impact beyond infection status alone. These results may be more broadly the case in other amphibian species and systems affected by chytridiomycosis. Indeed, several other frog populations initially decimated by chytridiomycosis appear to have similarly experienced recoveries (Scheele et al., 2017), and several species feared extinct have been rediscovered in the past decade (e.g., Amorós et al., 2020). Our findings yield promise for the recovery of numerous amphibian species globally in the face of endemic *Bd*. However, it is concerning and important to note that *Bd* remains a potential threat even in recovered populations, with the capacity to cause mortality at high infection loads, particularly if environmental conditions change, or in synergy with other threatening processes.

## AUTHOR CONTRIBUTIONS

David A. Newell designed the study, and David A. Newell and Matthijs Hollanders collected the data; Matthijs Hollanders, Catherine J. Nock, and Laura F. Grogan performed laboratory work; Matthijs Hollanders performed statistical analysis; and Matthijs Hollanders wrote the first draft of the paper. All authors contributed critically to the drafts and gave final approval for publication.

## CONFLICT OF INTEREST

The authors declare no conflict of interest.

## Supporting information


Appendix S1

Appendix S2

Appendix S3

## Data Availability

Data (Hollanders et al., 2022a) are available on Dryad at https://doi.org/10.5061/dryad.g1jwstqtb. Code (Hollanders et al., 2022b) is available on Zenodo at https://doi.org/10.5281/zenodo.6981761.
