# Supplementary material for: Recovered frog populations coexist with endemic Batrachochytrium dendrobatidis despite load‐dependent mortality
Source: Ecol Appl. 2022 Oct 27;33(1):e2724. doi: 10.1002/eap.2724 (PMC10078584; doi:10.1002/eap.2724)
Supplement: Supplementary file 1 — Appendix S1 [file EAP-33-0-s001.pdf]

Appendix S1: Supplementary methods

Matthijs Hollanders, Laura F. Grogan, Catherine J. Nock, Hamish I. McCallum, David A. Newell

Manuscript: Recovered frog populations coexist with endemic *Batrachochytrium dendrobatidis* despite load-dependent mortality
Journal: Ecological Applications

Table of contents

Laboratory protocol to detect and quantify *Bd* infections **2**

Mark-recapture modeling **3**
- Ecological process . . . 3
- Observation process . . . 4
- Priors . . . 4
- Reversible Jump MCMC . . . 5
- Imputation of missing values . . . 5
- Posterior predictive checks . . . 5

References **7**

Laboratory protocol to detect and quantify *Bd* infections

DNA was extracted from swab tips with Prepman® Ultra (Applied Biosystems) using a standard protocol (Boyle et al. 2004, Hyatt et al. 2007) without bead-beating step (Brannelly et al. 2020). Swab tips were cut off using scissors into 1.5 ml tubes containing 70 μl Prepman® Ultra. In order to avoid contamination, all tubes remained closed until respective swab tips were cut and scissors were cleaned with freshly made 2% bleach solution and rinsed with Milli-Q water between each sample. A negative control swab was included with every run of extractions to test for contamination, of which we observed no evidence. After cutting swab tips, tubes were briefly vortexed to ensure mixing with Prepman® Ultra and incubated at 100ºC for 10 min in an oven. Samples were allowed to cool for 2 min and centrifuged for 3 min at 13,000 rpm. Supernatant was recovered and 5 μl was diluted 10^{-1} with UltraPure Distilled Water (Invitrogen) for diagnosis using quantitative Polymerase Chain Reaction (qPCR).

We used a Rotor-Gene Q (Qiagen) to amplify and quantify *Bd* ITS copies for 40 cycles following modified standard protocols (Boyle et al. 2004). We used Rotor-Discs with 100 wells and 15 μl reaction volumes containing 7.5 μl (1X) Sensi-FAST™ Probe Lo-ROX Mix (Bioline), 900 nM primers (ITS1-3 5'-CCTTGATATAATACAGTGTGCCATATGTC-3' and 5.8S 5'-AGCCAAGAGATCCGTTGTCAAA-3'; Sigma-Aldrich), 250 nM TaqMan™ ChytrMGB2 probe (6-FAM-CGAGTCGAACAAAAT-MGB-NFQ; ThermoFisher), 400 ng/μl UltraPure™ BSA (Invitrogen), Taqman™ Exogenous Internal Positive Controls (with 1.5 μl 10X Mix and 0.3 μl 50X DNA), and 5 μl template. Included in each run were five reactions containing gBlocks® synthetic ITS standards (Integrated DNA Technologies) increasing by orders of magnitude from 10^{1-5} copies, from which a standard curve was generated to quantify ITS copies in each well. Runs included three no template controls (NTCs, to test for contamination) with UltraPure™ Distilled Water instead of template.

Swab samples were run in duplicate and considered positive when at least one well amplified more than 0 ITS copies. Our assay consistently recovered small *Bd* loads which likely represent low infection levels (Hyatt et al. 2007, Briggs et al. 2010). We report infection intensity as $\log_{10}$ ITS copies per swab because the ITS copy number of local *Bd* strains is unknown (Longo et al. 2013). To generate ITS copies per swab, estimated numbers of ITS copies per well were averaged over the duplicate runs and multiplied by the dilution factors in the extraction process.

Mark-recapture modeling

We fit a robust design, multisite, multistate Arnason-Schwarz (Arnason 1972, 1973, Schwarz et al. 1993, Kéry and Schaub 2012) using a continuous-time formulation of the ecological process (Miller and Andersen 2008, Conn et al. 2012, Glennie et al. 2022). Our robust design consisted of 19–21 primary occasions per site with 2–3 secondary surveys per primary occasion per site. We modeled the ecological process with hazard rates and matrix exponentials to account for unequal time intervals during the first two years of the study, and because this method is more appropriate for modeling processes occurring instantaneously in continuous-time (Ergon et al. 2018). We specified six-weeks as the baseline interval between primary occasions—note that this implicitly assumes that infection state transitions were not expected to occur more than once over six-weekly time periods. We conducted our analysis with NIMBLE 0.12.2 (de Valpine et al. 2017) in R 4.1.0 (R Core Team 2021).

Ecological process

We modeled three latent ecological states (z): (1) alive and *Bd*–, (2) alive and *Bd*+, and (3) dead. Starting after an individual's first capture, the latent ecological state of individual i during primary occasion t at site m was modeled conditional on the ecological state during primary occasion $t-1$:

$$z_{itm} | z_{it-1m} \sim \text{Categorical}(\mathbf{TPM_z}) \tag{S1}$$

where the transition *probability* matrix (TPM) of the ecological process $\mathbf{TPM_z} = e^{\mathbf{TRM_z}\tau}$, where τ is the vector of site-specific primary occasion intervals in units of six-weeks and $\mathbf{TRM_z}$ is the transition *rate* matrix (TRM) of the ecological process (Miller and Andersen 2008, Conn et al. 2012):

$$\begin{array}{cc} & z_{itm} \\ & \begin{array}{ccc} \text{Alive, } Bd\text{–} & \text{Alive, } Bd\text{+} & \text{Dead} \end{array} \\ z_{it-1m} \begin{array}{r} \text{Alive, } Bd\text{–} \\ \text{Alive, } Bd\text{+} \\ \text{Dead} \end{array} & \begin{bmatrix} -(\psi_{12} + \phi_1) & \psi_{12} & \phi_1 \\ \psi_{21} & -(\psi_{21} + \phi_2) & \phi_2 \\ 0 & 0 & 0 \end{bmatrix}_{itm} \end{array}$$

Above, ϕ_1 is the mortality hazard rate of uninfected individuals, ϕ_2 is the mortality hazard rate of infected individuals, ψ_{12} is the hazard rate of gaining *Bd* infection, and ψ_{21} is the hazard rate of clearing *Bd* infection. We calculated $e^{\mathbf{TRM_z}} = \mathbf{V}e^{\mathbf{D}\tau}\mathbf{V^{-1}}$, where $\mathbf{V}$ is the matrix of eigenvectors of $\mathbf{TRM_z}$ and $\mathbf{D}$ is the diagonal matrix of eigenvalues of $\mathbf{TRM_z}$ (Miller and Andersen 2008, Conn et al. 2012). Note that when using NIMBLE, one can use `nimbleRcall` to call the `expm::expm()` function within the model code (Goulet et al. 2021), but this approach is approximately 10 times slower than using NIMBLE's built-in matrix functions.

All hazard rates were modeled at the level of site-specific primary occasions. We modeled mortality hazard rates as log-linear functions of body condition (scaled mass index, Peig and Green 2009), average daily temperature over the primary interval (a proxy for season), *Bd* infection status (ϕ_2 only), *Bd* infection intensity (ϕ_2 only), and all pairwise interactions. After standardizing predictors,

including *Bd* infection intensity, the effect of *Bd* infection status can be interpreted as the effect of being infected with *Bd* with an average infection intensity on the log mortality hazard rate. Hazard rates of infection transitions were modeled as log-linear functions of body condition, average daily temperature over the primary interval, average six-weekly rainfall over the primary interval, their interaction, and *Bd* infection intensity (ψ_{21} only). All mortality and infection transition parameters additionally included random site and survey effects, which were drawn from two multivariate normal distributions, respectively. Note that the mortality hazard rates are 'apparent' mortality hazard rates, as mortality could not be disentangled from permanent emigration from the sites.

Observation process

The observation process was modeled conditional on the latent ecological state, with three possible observed states (y, data): (1) seen/recaptured and *Bd*–, (2) seen/recaptured and *Bd*+, and (3) not seen/recaptured. Note that the observed state was assigned based on the collected swab samples. The observed state of individual i, primary occasion t, secondary survey k, at site m was drawn from a categorical distribution conditional on the latent ecological state:

$$y_{itkm}|z_{itm} \sim \text{Categorical}(\mathbf{TPM_o}) \tag{S2}$$

Where the TPM of the observation process ($\mathbf{TPM_o}$) was:

$$z_{itm} \quad \begin{array}{r} \\ \\ \text{Alive, } Bd\text{–} \\ \text{Alive, } Bd\text{+} \\ \text{Dead} \end{array} \begin{array}{ccc} & y_{itkm} & \\ \text{Seen, } Bd\text{–} & \text{Seen, } Bd\text{+} & \text{Not seen} \\ \end{array} \left[\begin{array}{ccc} p_1 & 0 & 1-p_1 \\ 0 & p_2 & 1-p_2 \\ 0 & 0 & 1 \end{array} \right]_{itkm}$$

where p_1 and p_2 are the recapture probabilities of individuals uninfected and infected with *Bd*, respectively. We modeled recapture probabilities at the level of secondary surveys as logit-linear functions of body condition, sex, daily temperature on the survey day, cumulative rainfall over the primary interval, their interaction, and *Bd* infection status and intensity (p_2 only). We additionally included random site effects, random secondary-survey level effects, and random individual effects. We included the individual effects to account for heterogeneity due to differences in individual home ranges, transience, and position on the transect.

Priors

For hazard rates, we used Exponential(1) for the log intercepts of log-linear functions because this corresponds to a uniform distribution on the baseline hazard rates transformed to probabilities. For recapture probabilities, we used Beta$(1, 1)$ for the logit intercept because this is a uniform distribution on the baseline recapture probabilities. We used weakly informative (Half-)Cauchy$(0, 2.5)$ on all predictor variables and standard deviations of random effects (Gelman et al. 2008).

Reversible Jump MCMC

For predictor variable selection, we used NIMBLE's built-in reversible jump MCMC (RJMCMC, Green 1995). RJMCMC samples across models with different dimensionalities (e.g., more or fewer predictors), and readily facilitates model selection directly within the MCMC algorithms. When RJMCMC includes a predictor in the model, the coefficients are estimated; when RJMCMC excludes a predictor, the coefficient is toggled to 0. After running the MCMC, the inclusion probability of predictors can be retrieved by calculating the proportion of MCMC samples for which a predictor was included in the model. Important predictors will be included in nearly all iterations, and predictors with little predictive potential will be excluded. We constrained interaction effects to only be included in the presence of main effects to maintain the principle of marginality.

Imputation of missing values

NIMBLE does not allow missing values in predictor variables. Therefore, for predictors with missing values, in particular matrices of time-varying individual covariates, missing values need to be provided. Bayesian analysis readily facilitates the imputation of these missing values using submodels for the covariates (Gelman et al. 2013). We imputed missing values in three predictor variables: body condition, infection intensity, and sex. For body condition, we imputed missing values from a normal distribution of the observed body condition values with random individual effects to account for repeat measures over the study. For infection intensity, we similarly imputed missing values from a normal distribution with random individual effects, but also included temperature, rainfall, and their interaction to account for seasonal differences in infection intensity. For sex, we imputed 15 missing values from a Bernoulli distribution roughly centered on the observed sex ratio.

Posterior predictive checks

We assessed goodness-of-fit through posterior predictive checks (PPCs, Gelman et al. 1996) inspired by Rankin et al. (2016) and Kéry and Royle (2020). PPCs are conducted by simulating replicate datasets (y^{rep}) from the joint posterior distribution for each MCMC iteration, calculating some summary statistics for the observed and replicate datasets, and then comparing some discrepancy statistics between observed and replicated datasets. By comparing the discrepancy statistics from the data (D) with the discrepancy statistics from the replicate datasets (D^{rep}), we assess to what extent our model predicts capture histories that are consistent with our observed data. Bayesian p-values summarize the similarities between observed and replicated data, calculated as $\Pr(D^{\text{rep}} > D)$, where values around 0.5 imply good fit. Note that our PPCs exclude the primary occasion of first capture, because this occasion is not modeled and thus not simulated in the replicate datasets.

For the first PPC, we counted the number of observed infection state transitions (going from uninfected to infected and vice versa) for each individual in the study. Note that infection state transitions can only be observed if individuals are observed during consecutive primaries in different states. We then calculated Freeman-Tukey statistics on these observed state transitions to calculate a Bayesian p-value. This PPC was conducted to assess the fit of the ecological process.

For the second PPC, we counted the number of times each individual was observed as either uninfected or infected with *Bd* during a secondary survey. Our summary statistics were thus the total number of recaptures of each individual for each alive state. We computed Freeman-Tukey

statistics and calculated Bayesian p-values. This PPC was conducted to assess the fit of the observation process.

References

Arnason, A. N. 1972. Parameter estimates from mark-recapture experiments on two populations subject to migration and death. Researches on Population Ecology 13:97–113.

Arnason, A. N. 1973. The estimation of population size, migration rates and survival in a stratified population. Researches on Population Ecology 15:1–8.

Boyle, D. G., D. B. Boyle, V. Olsen, J. A. T. Morgan, and A. D. Hyatt. 2004. Rapid quantitative detection of chytridiomycosis (*Batrachochytrium dendrobatidis*) in amphibian samples using real-time Taqman PCR assay. Diseases of Aquatic Organisms 60:141–148.

Brannelly, L. A., D. P. Wetzel, M. West, and C. L. Richards-Zawacki. 2020. Optimized *Batrachochytrium dendrobatidis* DNA extraction of swab samples results in imperfect detection particularly when infection intensities are low. Diseases of Aquatic Organisms 139:233–243.

Briggs, C. J., R. A. Knapp, and V. T. Vredenburg. 2010. Enzootic and epizootic dynamics of the chytrid fungal pathogen of amphibians. Proceedings of the National Academy of Sciences 107:9695–9700.

Conn, P. B., E. G. Cooch, and P. Caley. 2012. Accounting for detection probability when estimating force-of-infection from animal encounter data. Journal of Ornithology 152:511–520.

de Valpine, P., D. Turek, C. J. Paciorek, C. Anderson-Bergman, D. T. Lang, and R. Bodik. 2017. Programming with models: Writing statistical algorithms for general model structures with NIMBLE. Journal of Computational and Graphical Statistics 26:403–413.

Ergon, T., Ø. Borgan, C. R. Nater, and Y. Vindenes. 2018. The utility of mortality hazard rates in population analyses. Methods in Ecology and Evolution 9:2046–2056.

Gelman, A., J. B. Carlin, H. S. Stern, D. B. Dunson, A. Vehtari, and D. B. Rubin. 2013. Chapter 18. Models for missing data. Bayesian Data Analysis. Third. CRC Press, Boca Raton, FL, USA.

Gelman, A., A. Jakulin, M. G. Pittau, and Y.-S. Su. 2008. A weakly informative default prior distribution for logistic and other regression models. The Annals of Applied Statistics 2:1360–1383.

Gelman, A., X.-L. Meng, and H. Stern. 1996. Posterior predictive assessment of model fitness via realized discrepancies. Statistica Sinica 6:733–807.

Glennie, R., T. Adam, V. Leos-Barajas, T. Michelot, T. Photopoulou, and B. T. McClintock. 2022. Hidden Markov models: Pitfalls and opportunities in ecology. Methods in Ecology and Evolution:2041–210X.13801.

Goulet, V., C. Dutang, M. Maechler, D. Firth, M. Shapira, and M. Stadelmann. 2021. Package 'expm'.

Green, P. J. 1995. Reversible Jump Markov Chain Monte Carlo computation and Bayesian model determination. Biometrika 82:711–732.

Hyatt, A. D., D. G. Boyle, V. Olsen, D. B. Boyle, L. Berger, D. Obendorf, A. Dalton, K. Kriger, M. Hero, H. Hines, R. Phillott, R. Campbell, G. Marantelli, F. Gleason, and A. Colling. 2007. Diagnostic assays and sampling protocols for the detection of *Batrachochytrium dendrobatidis*. Diseases of Aquatic Organisms 73:175–192.

Kéry, M., and J. A. Royle. 2020. Chapter 4: Modeling species distribution and range dynamics, and population dynamics. Applied Hierarchical Modeling in Ecology: Analysis of Distribution, Abundance and Species Richness in R and BUGS: Volume 2: Dynamic and Advanced Models. Academic Press.

Kéry, M., and M. Schaub. 2012. Chapter 9. Estimation of survival and movement from capture-recapture data using multistate models. Pages 264–315 Bayesian Population Analysis Using WinBUGS: A Hierarchical Perspective. Academic Press.

Longo, A. V., D. Rodriguez, D. da Silva Leite, L. F. Toledo, C. Mendoza Almeralla, P. A. Burrowes, and K. R. Zamudio. 2013. ITS1 copy number varies among *Batrachochytrium dendrobatidis* strains: Implications for qPCR estimates of infection intensity from field-collected amphibian skin swabs. PLoS ONE 8:e59499.

Miller, T. J., and P. K. Andersen. 2008. A finite-state continuous-time approach for inferring regional migration and mortality rates from archival tagging and conventional tag-recovery experiments. Biometrics 64:1196–1206.

Peig, J., and A. J. Green. 2009. New perspectives for estimating body condition from mass/length data: The scaled mass index as an alternative method. Oikos 118:1883–1891.

R Core Team. 2021. R: A language and environment for statistical computing. R Foundation for Statistical Computing, Vienna, Austria.

Rankin, R. W., K. E. Nicholson, S. J. Allen, M. Krützen, L. Bejder, and K. H. Pollock. 2016. A full-capture hierarchical Bayesian model of Pollock's closed robust design and application to dolphins. Frontiers in Marine Science 3.

Schwarz, C. J., J. F. Schweigert, and A. N. Arnason. 1993. Estimating migration rates using tag-recovery data. Biometrics 49:177–193.
