# Supplementary material for: Recovered frog populations coexist with endemic Batrachochytrium dendrobatidis despite load‐dependent mortality
Source: Ecol Appl. 2022 Oct 27;33(1):e2724. doi: 10.1002/eap.2724 (PMC10078584; doi:10.1002/eap.2724)
Supplement: Supplementary file 2 — Appendix S2 [file EAP-33-0-s002.pdf]

Appendix S2: Results and discussion of recapture probabilities

Matthijs Hollanders, Laura F. Grogan, Catherine J. Nock, Hamish I. McCallum, David A. Newell

Manuscript: Recovered frog populations coexist with endemic *Batrachochytrium dendrobatidis* despite load-dependent mortality
Journal: Ecological Applications

Table of contents

Results 2

Discussion 2

References 3

Results

Average site-specific recapture probabilities ranged from 0.14–0.219 (Appendix S2: Table S2). Body condition significantly predicted recapture probability (log odds change 0.839 [0.451, 1.208], *pd* = 100%, 0% in ROPE, 99.9% inclusion), and females had significantly lower recapture probability compared to males (log odds change -3.443 [-4.177, -2.738], *pd* = 100%, 0% in ROPE, 100% inclusion) (Appendix S2: Table S2 and Figure S3a). Detection probabilities peaked in summer and dipped in winter (Figure 3d, Appendix S2: Table S2 and Figure S2d). Daily temperature (log odds change 1.298 [0.809, 1.772], *pd* = 100%, 0% in ROPE, 100% inclusion) and interval rainfall (log odds change 0.59 [0.092, 1.067], *pd* = 99.1%, 2.1% in ROPE, 99.1% inclusion) significantly increased recapture probability (Appendix S2: Table S2 and Figure S3b). *Bd* infection status positively correlated with recapture (log odds change 0.805 [0.215, 1.348], *pd* = 99.3%, 1.2% in ROPE, 98.1% inclusion) (Appendix S2: Table S2 and Figure S3a), but infection intensity did not (log odds change 0.092 [-0.827, 0.95], *pd* = 58.3%, 73.3% inclusion). Unexplained temporal variation was fairly high (SD of random survey effect on log odds 0.929 [1.287, 1.771]) and there was substantial individual variation (SD on log odds 1.523 [1.287, 1.771]).

Discussion

Activity patterns of *M. fleayi*, described in our analysis by the recapture probability of marked animals, were significantly predicted by sex, individual body condition, temperature and rainfall, and *Bd* infection status. Females were much less likely to be recaptured than males, likely reflecting the observation that females spend considerable time away from streams and probably only visit them to breed. Increased individual body condition was strongly associated with recapture, which, to our knowledge, has not previously been reported in literature. Increased body condition may increase activity of frogs, or these individuals may claim stream-side territories, increasing recaptures along stream transects. As expected, temperature and rainfall were positively associated with recapture. Average rainfall over the preceding six weeks had a greater influence on activity than rainfall on the survey day, which did not correlate with detection. We found evidence that frogs infected with *Bd* were more likely to be recaptured than uninfected frogs, a result that has been found in some previous capture-recapture studies (Heard et al. 2014, Russell et al. 2019) but not others (Murray et al. 2009, Briggs et al. 2010, Grogan et al. 2016, Muths et al. 2020). Our results may be confounded with distance from the stream, where individuals residing closer to the water may be more likely to be infected with *Bd*, though studies relating infection patterns to stream distance are lacking. It is further possible that infected frogs are more active, as previous studies have found that *Bd* infection influences host life history traits, specifically mating behavior (e.g., Kelleher et al. 2021). Increased detection of frogs would be expected if males were investing more energy into reproductive behavior when infected, especially given that a large proportion of detections were the result of calling. We did not investigate the effect of *Bd* infection on calling behavior, but future capture-recapture studies may record whether frogs were calling upon capture. Unlike Heard et al. (2014), we found no evidence of *Bd* infection intensity influencing recapture probability. This result suggests that increased recapture probability of infected individuals is not an effect of infection pathology, as behavioral effects of *Bd* infection would be expected to increase with infection intensity.

References

Briggs, C. J., R. A. Knapp, and V. T. Vredenburg. 2010. Enzootic and epizootic dynamics of the chytrid fungal pathogen of amphibians. Proceedings of the National Academy of Sciences 107:9695–9700.

Grogan, L. F., A. D. Phillott, B. C. Scheele, L. Berger, S. D. Cashins, S. C. Bell, R. Puschendorf, and L. F. Skerratt. 2016. Endemicity of chytridiomycosis features pathogen overdispersion. Journal of Animal Ecology 85:806–816.

Heard, G. W., M. P. Scroggie, N. Clemann, and D. S. L. Ramsey. 2014. Wetland characteristics influence disease risk for a threatened amphibian. Ecological Applications 24:650–662.

Kelleher, S. R., B. C. Scheele, A. J. Silla, J. S. Keogh, D. A. Hunter, J. A. Endler, and P. G. Byrne. 2021. Disease influences male advertisement and mating outcomes in a critically endangered amphibian. Animal Behaviour 173:145–157.

Murray, K. A., L. F. Skerratt, R. Speare, and H. McCallum. 2009. Impact and dynamics of disease in species threatened by the amphibian chytrid fungus, *Batrachochytrium dendrobatidis*. Conservation Biology 23:1242–1252.

Muths, E., B. R. Hossack, E. H. Campbell Grant, D. S. Pilliod, and B. A. Mosher. 2020. Effects of snowpack, temperature, and disease on demography in a wild population of amphibians. Herpetologica 76:132–143.

Russell, R. E., B. J. Halstead, B. A. Mosher, E. Muths, M. J. Adams, E. H. C. Grant, R. N. Fisher, P. M. Kleeman, A. R. Backlin, C. A. Pearl, R. K. Honeycutt, and B. R. Hossack. 2019. Effect of amphibian chytrid fungus (*Batrachochytrium dendrobatidis*) on apparent survival of frogs and toads in the western USA. Biological Conservation 236:296–304.
