# Supplementary material for: Recovered frog populations coexist with endemic Batrachochytrium dendrobatidis despite load‐dependent mortality
Source: Ecol Appl. 2022 Oct 27;33(1):e2724. doi: 10.1002/eap.2724 (PMC10078584; doi:10.1002/eap.2724)
Supplement: Supplementary file 3 — Appendix S3 [file EAP-33-0-s003.pdf]

Appendix S3: Supplementary tables and figures

Matthijs Hollanders, Laura F. Grogan, Catherine J. Nock, Hamish I. McCallum, David A. Newell

Manuscript: Recovered frog populations coexist with endemic *Batrachochytrium dendrobatidis* despite load-dependent mortality
Journal: Ecological Applications

Table of contents

Supplementary tables 2

Supplementary figures 5

References 8

Supplementary tables

Table S1: Estimates from 10,000 posterior samples of the logit-linear *Bd* infection prevalence and log-linear *Bd* intensity models. Species-level intercepts are reported on the original scale (probability for prevalence and $\log_{10}$ ITS copies for intensity, respectively). Coefficients for predictors on prevalence and intensity are log odds and log-linear, respectively. Random site, individual, and temporal effects are reported as standard deviations of the variation's distribution on the scale of the link function. Stars indicate effects that are likely to exist ($pd > 97\%$), and bold face indicates 'significant' predictors for which the percentage of the full posterior distribution in the Region of Practical Equivalence (ROPE) was < 2.5% (Makowski et al. 2019). RJMCMC (%) is the inclusion probability of the effect in the model (Green 1995). Species-by-site level estimates are plotted in Fig. 1a–b.

**Model**	**Parameter**	**Median**	**95% HDI**	***pd* (%)**	**ROPE (%)**	**RJMCMC (%)**
Prevalence						
	Litoria pearsoniana	0.229	[0.112, 0.384]			
	Litoria wilcoxii	0.223	[0.098, 0.401]			
	Mixophyes iteratus	0.575	[0.423, 0.725]			
	Mixophyes fleayi	0.152	[0.045, 0.243]			
	**Temperature***	-1.025	[-1.631, -0.449]	100	0.1	93.4
	**Rainfall***	0.75	[0.212, 1.314]	99.5	1	86.7
	Temperature × rainfall	0.018	[-1.21, 1.162]	51.1	54.7	22.3
	Site effect	0.371	[0.007, 1.08]			
	Individual effect	0.531	[0.011, 0.91]			
	Temporal effect	0.667	[0.443, 0.933]			
Intensity						
	Litoria pearsoniana	4.014	[3.636, 4.402]			
	Litoria wilcoxii	3.748	[3.336, 4.174]			
	Mixophyes iteratus	3.614	[3.287, 3.949]			
	Mixophyes fleayi	3.036	[2.83, 3.228]			
	**Temperature***	-0.163	[-0.247, -0.089]	100	0.1	98.9
	Rainfall*	0.078	[0.013, 0.143]	98.5	20	26.2
	Temperature × rainfall					2.6
	Site effect	0.025	[0, 0.091]			
	Individual effect	0.112	[0.039, 0.179]			
	Temporal effect	0.055	[0.009, 0.099]			
	Residual	0.243	[0.204, 0.275]			

Table S2: Estimates for the apparent mortality rates, rates of gaining and clearing *Bd* infection, and recapture probabilities of *M. fleayi* from 10,000 posterior samples of the robust design multistate Arnason-Schwarz model. Site-level intercepts are reported as hazard rates for apparent mortality and state transitions and as probabilities for recapture; coefficients and random effects are on the scale of the link function (log for hazard rates and logit for recapture). Random temporal and individual effects are reported as standard deviations of the variation's distribution. Stars indicate effects that are likely to exist ($pd > 97\%$), and bold face indicates 'significant' predictors for which the percentage of the full posterior distribution in the Region of Practical Equivalence (ROPE) was $< 2.5\%$ (Makowski et al. 2019). RJMCMC (%) is the inclusion probability of the effect in the model (Green 1995).

**Function**	**Parameter**	**Median**	**95% HDI**	***pd* (%)**	**ROPE (%)**	**RJMCMC (%)**
Mortality						
	Intercept (Brindle)	0.104	[0.07, 0.139]			
	Intercept (Tuntable)	0.077	[0.05, 0.108]			
	Intercept (Bat Cave)	0.092	[0.06, 0.128]			
	Body condition*	0.414	[0.002, 0.823]	97.2	4.7	97.8
	Temperature	0.176	[-0.393, 0.686]	73.3	32.5	97.3
	Bd status	-0.167	[-1.714, 1.053]	60	36.5	79.4
	Bd intensity*	1.953	[-0.011, 3.834]	97.5	2.8	99.9
	Body condition × temperature	0.801	[-0.076, 1.563]	96.7	4.1	88.9
	Body condition × *Bd* intensity	0.156	[-1.569, 2.034]	58.5	44.2	81.4
	Temperature × *Bd* intensity	-0.084	[-2.311, 2.118]	53.4	44.5	82.3
	Temporal effect	0.334	[0, 0.7]			
Gaining *Bd*						
	Intercept (Brindle)	0.244	[0.094, 0.497]			
	Intercept (Tuntable)	0.564	[0.172, 1.404]			
	Intercept (Bat Cave)	0.163	[0.021, 0.497]			
	Body condition	0.138	[-0.703, 0.912]	63.4	41.6	75.1
	Temperature	-0.419	[-1.61, 0.79]	76.4	26.5	93.2
	**Rainfall***	1.141	[-0.013, 2.253]	98.1	2.5	98.7
	Temperature × rainfall	0.079	[-1.602, 1.608]	54.1	48.5	76.1
	Temporal effect	0.492	[0.004, 0.981]			
Clearing *Bd*						
	Intercept (Brindle)	2.53	[1.271, 4.721]			
	Intercept (Tuntable)	2.892	[1.159, 7.037]			
	Intercept (Bat Cave)	2.418	[0.492, 6.895]			
	Body condition	-0.392	[-1.201, 0.351]	85.3	17.5	81.1
	Temperature	1.101	[-0.094, 2.302]	96.4	4.2	98.5
	Rainfall	-0.246	[-1.42, 0.797]	67.8	35.6	92.4
	Bd intensity	0.297	[-0.407, 1.024]	80.3	24.1	75.6
	Temperature × rainfall	-0.467	[-2.192, 1.154]	71.9	30.2	78.8
	Temporal effect	0.29	[0, 0.786]			
Recapture						

**Function**	**Parameter**	**Median**	**95% HDI**	***pd (%)***	**ROPE (%)**	**RJMCMC (%)**
	Intercept (Brindle)	0.14	[0.092, 0.192]			
	Intercept (Tuntable)	0.219	[0.132, 0.313]			
	Intercept (Bat Cave)	0.182	[0.103, 0.29]			
	**Body condition***	0.839	[0.451, 1.208]	100	0	99.9
	**Sex (female)***	-3.443	[-4.177, -2.738]	100	0	100
	**Temperature***	1.298	[0.809, 1.772]	100	0	100
	**Rainfall***	0.59	[0.092, 1.067]	99.1	2.1	99.1
	Bd* status	0.805	[0.215, 1.348]	99.3	1.2	98.1
	Bd intensity	0.092	[-0.827, 0.95]	58.3	49.9	73.3
	Temperature × rainfall	0.175	[-0.863, 1.365]	37.8	68	77.9
	Temporal effect	0.929	[0.768, 1.111]			
	Individual effect	1.523	[1.287, 1.771]			

Supplementary figures

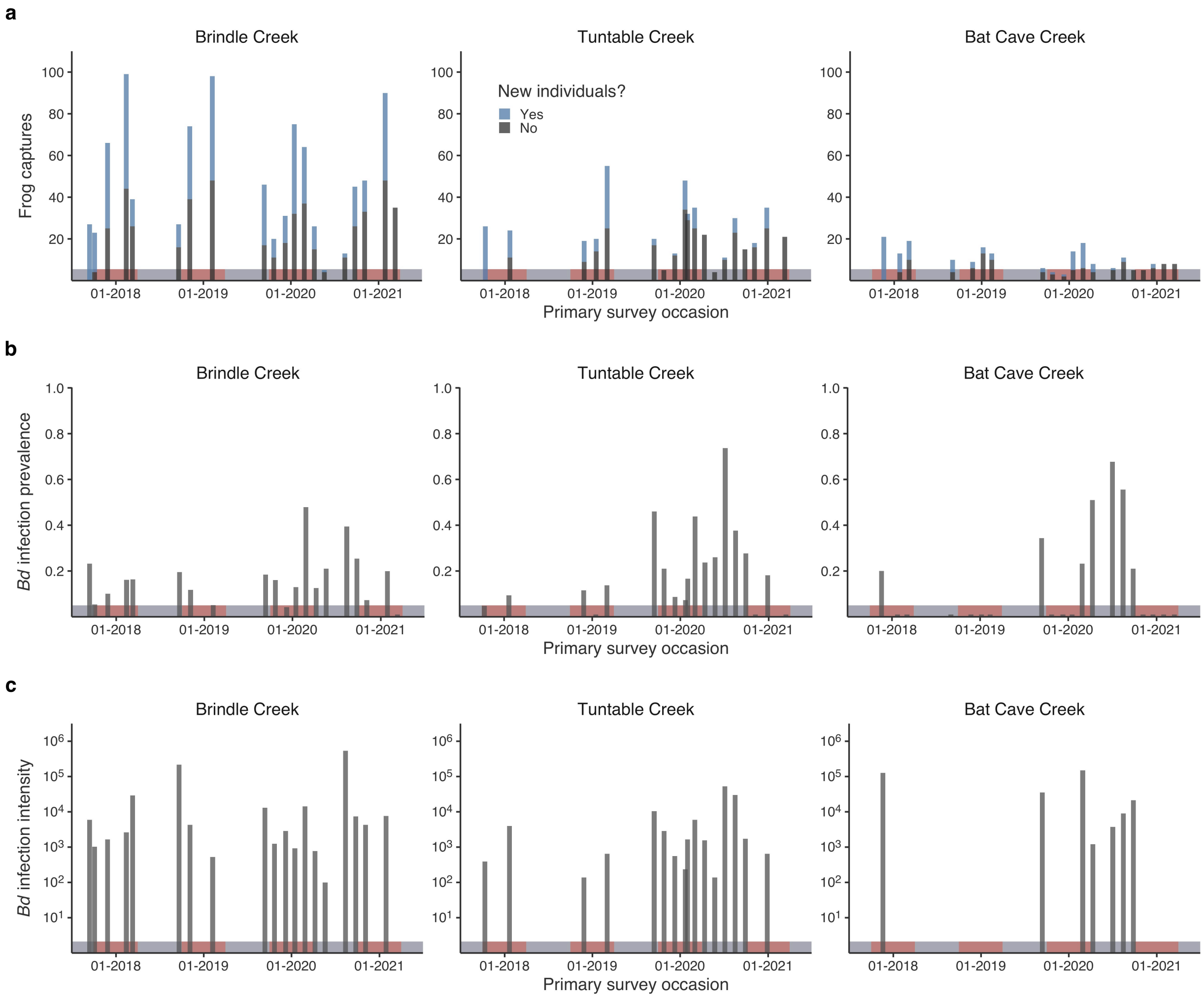

Figure S1: Site-specific *M. fleayi* (**a**) frog captures, (**b**) *Bd* infection prevalence, and (**c**) *Bd* infection intensity (ITS copies per swab) per primary occasion. Observed frog captures are split between new and previously marked animals.

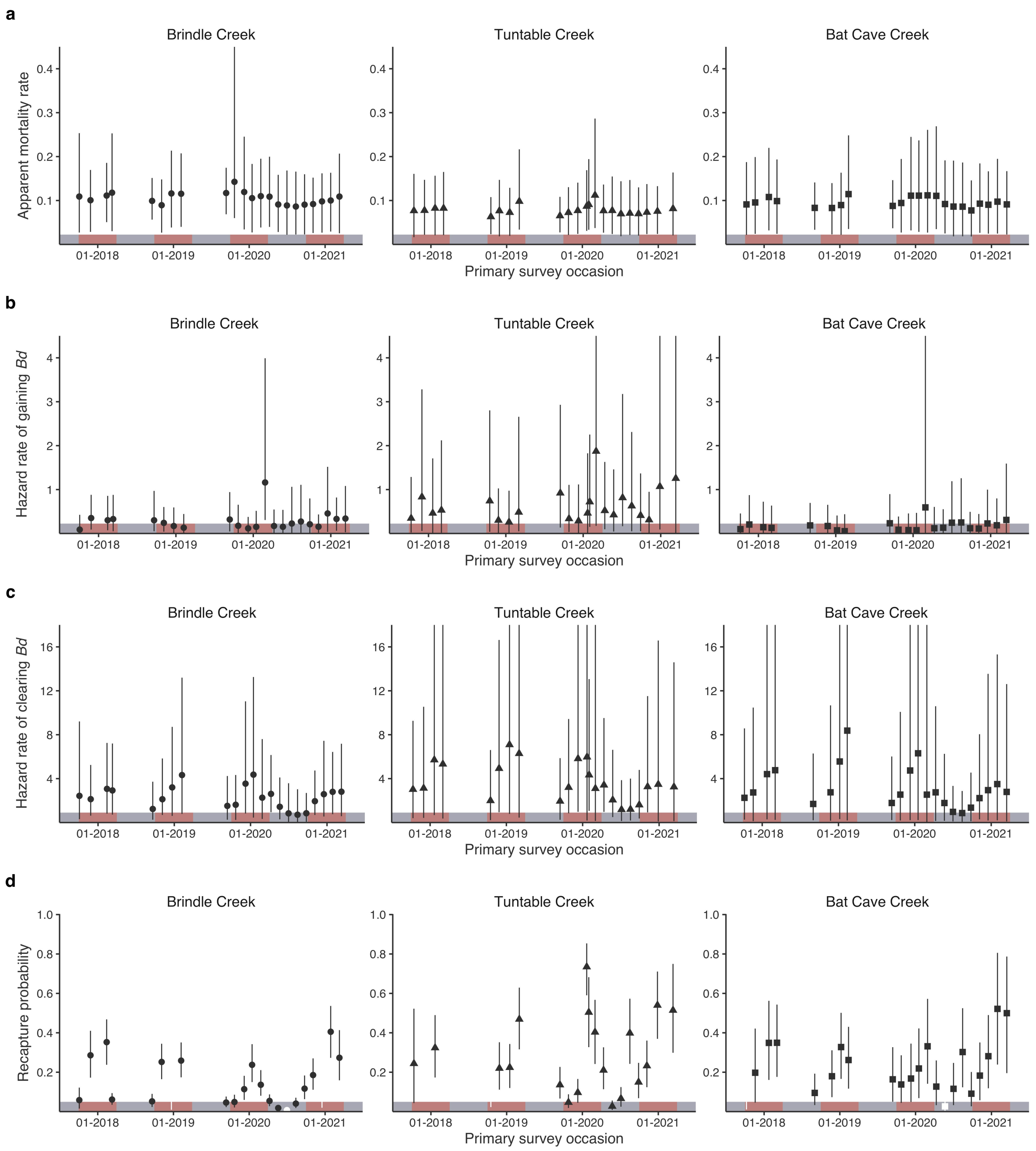

Figure S2: Estimates (median and 95% HDI) of site-specific *M. fleayi* (**a**) six-weekly apparent mortality rate, (**b**) six-weekly hazard rate of gaining *Bd* infection, (**c**) six-weekly hazard rate of clearing *Bd* infection, and (**d**) recapture probability per primary occasion. Red and grey demarcations indicate austral summer (October–April) and winter (May–September), respectively.

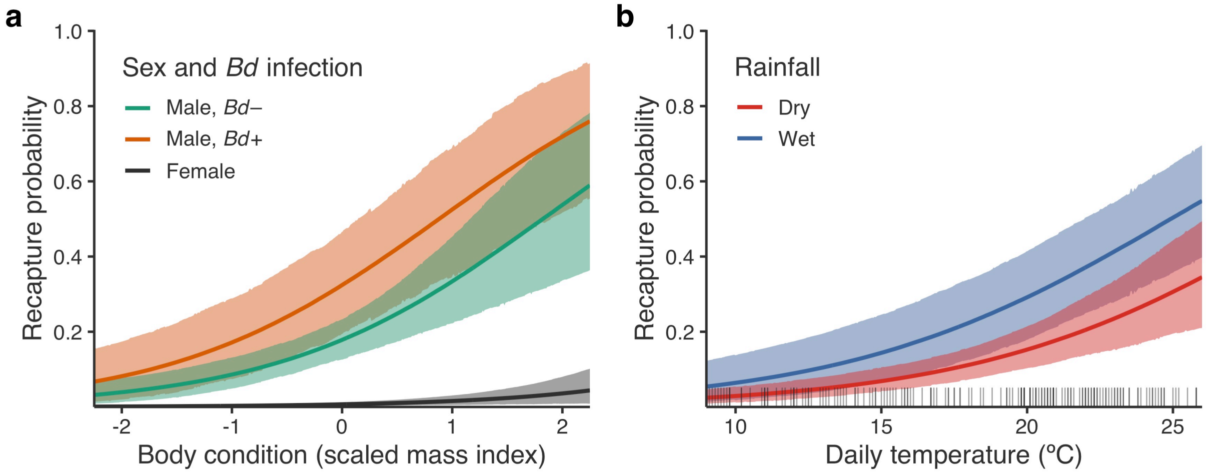

Figure S3: Predictions of the effects of (**a**) body condition (scaled mass index), sex, and *Bd* infection status and (**b**) daily temperature and cumulative rainfall on recapture probability of *M. fleayi*. Scaled mass index is presented as centered and standardized by two standard deviations. Dry and wet rainfall periods correspond to no rainfall and 575 mm (approximately two standard deviations above the observed mean of 195 mm) over six-week survey intervals. The intercepts of the curves are the means of the site-specific intercepts. The rug plot in (**b**) shows observed daily temperature per secondary survey day.

References

Green, P. J. 1995. Reversible Jump Markov Chain Monte Carlo computation and Bayesian model determination. Biometrika 82:711–732.

Kay, M. 2021. ggdist: Visualizations of distributions and uncertainty, R package version 2.2.0.

Makowski, D., M. S. Ben-Shachar, S. H. A. Chen, and D. Lüdecke. 2019. Indices of effect existence and significance in the Bayesian framework. Frontiers in Psychology 10:2767.

Wickham, H. 2016. ggplot2: Elegant graphics for data analysis. Springer-Verlag New York.
